# Complete Genome Sequence of a VIM-1-Producing Salmonella enterica subsp. enterica Serovar Infantis Isolate Derived from Minced Pork Meat

**DOI:** 10.1128/genomeA.00327-18

**Published:** 2018-04-26

**Authors:** Maria Borowiak, Jennie Fischer, Beatrice Baumann, Jens A. Hammerl, Istvan Szabo, Burkhard Malorny

**Affiliations:** a German Federal Institute for Risk Assessment (BfR), Department for Biological Safety, Berlin, Germany

## Abstract

Carbapenems are considered last-resort antibiotics used to treat human infections caused by multidrug-resistant bacteria. In 2011, VIM-1 carbapenemase-producing Salmonella enterica subsp. enterica serovar Infantis strains were isolated from livestock for the first time in Germany. Here, we announce the complete genome sequence of the first German *bla*_VIM-1_-harboring Salmonella Infantis isolate (15-SA01028) originating from food.

## GENOME ANNOUNCEMENT

Salmonella enterica subsp. enterica serovar Infantis is an important zoonotic pathogen causing gastroenteritis in humans. This pathogen is commonly transferred via contaminated food products (1). In Germany, S. Infantis is ranked as the third most common cause of human salmonellosis (2). In 2011, VIM-1 carbapenemase-producing S. Infantis isolates were detected in livestock farms in Germany (3, 4). As carbapenems are considered last-line clinical antibiotics used to treat severe human infections, these findings raised concerns on the potential spread of carbapenemase-producing isolates from animal settings via the food chain to the consumer.

In 2015, the National Reference Laboratory for Salmonella in Germany indeed received an S. Infantis strain isolated from minced pork meat within the framework of routine diagnostics. Antimicrobial susceptibility testing of this strain (15-SA01028) showed microbiological resistance to meropenem (MIC, 0.5 mg/liter), imipenem (MIC, 4 mg/liter), and ertapenem (MIC, 0.12 mg/liter) using the microdilution method (CLSI guideline CLSI M07-A9 [5]) and following EUCAST epidemiological cutoff values (ECOFFs) (http://www.eucast.org) (2).

Subsequent whole-genome sequencing (WGS) using Illumina MiSeq technology and S1-pulsed-field gel electrophoresis (S1-PFGE) hybridization of the respective isolate revealed that the isolate harbored a pRH-R27-like plasmid carrying a *bla*_VIM-1_-encoding carbapenemase (2). To understand the microevolution of the *bla*_VIM-1_-carrying plasmid and its transmission routes along the German food production system, the isolate was further subjected to PacBio RS II long-read sequencing.

Therefore, genomic DNA of isolate 15-SA01028 was extracted using the PureLink genomic DNA minikit (Invitrogen, Carlsbad, CA, USA). Genome sequencing was performed by GATC Biotech AG (Constance, Germany) using the PacBio RS II system. A *de novo* genome assembly was performed based on 32,727 reads from one single-molecule real-time (SMRT) cell (mean read length, 12,946 bp; *N*_50_ read length, 17,965 bp; mean read score, 0.85) using the SMRT Analysis software (version 2.3.0; Pacific Biosciences, USA) and resulted in two contigs with an average coverage of 75.27 per consensus base. Illumina paired-end short-read data were mapped against the PacBio reference genome using CLC Genomics Workbench 9.5.2. The contigs were closed to circular molecules, and errors in homopolymeric regions were corrected. The resulting genome consists of a bacterial chromosome (4,698,699 bp) and the *bla*_VIM-1_-carrying plasmid pSE15-SA01028 (310,921 bp), comprising average G+C contents of 52.3% and 47.7%, respectively.

Genome analysis using the ResFinder3.0 and PlasmidFinder1.3 tools provided by the Center for Genomic Epidemiology (http://www.genomicepidemiology.org) revealed that the plasmid pSE15-SA01028 belongs to the plasmid incompatibility group IncHI2A (sequence type 1 [ST1]) and harbors 12 antibiotic resistance genes. The *bla*_VIM-1_ gene was located on a Tn*21*-like transposon harboring two class 1 integrons containing, apart from the *sul1* gene, further resistance gene cassettes *aadA1-ere(A)* and *aadA1*-*aacA4*-*bla*_VIM-1_.

Genome annotation was performed using the automated Prokaryotic Genome Annotation Pipeline (https://www.ncbi.nlm.nih.gov/genome/annotation_prok/). The results revealed the presence of 5,029 coding sequences (CDS; 4,840 coding CDS and 189 pseudogenes), as well as 126 RNA genes (22 rRNAs, 87 tRNAs, and 17 noncoding RNAs) on the bacterial chromosome. With this sequence, we provide a high-quality reference for genome comparison studies.

### Accession number(s).

Sequences were deposited in GenBank under the accession numbers CP026660 (chromosome) and CP026661 (pSE15-SA01028).
